# Percutaneous left atrial appendage closure: the CHAMPION-like vs. CLOSURE-like patient phenotype framework

**DOI:** 10.1093/europace/euag286

**Published:** 2026-10-05

**Authors:** Sergio Berti, Philippe Garot, Giancarlo Trimarchi, Jens Erik Nielsen-Kudsk, Gregory Y H Lip, Carsten Skurk, Valeria Caso, Lucas V A Boersma, Domenico G Della Rocca, Giuseppe Boriani, Alan John Camm

**Affiliations:** Fondazione Toscana Gabriele Monasterio, Ospedale del Cuore ‘G. Pasquinucci’, Via Aurelia Sud, 54100 Massa, Italy; Interdisciplinary Center for Health Sciences, Scuola Superiore Sant’Anna, Piazza Martiri della Libertà, 33, 56127 Pisa, Italy; Institut Cardiovasculaire Paris Sud (ICPS), Hôpital Privé Jacques Cartier, Ramsay Santé, Massy, France; Fondazione Toscana Gabriele Monasterio, Ospedale del Cuore ‘G. Pasquinucci’, Via Aurelia Sud, 54100 Massa, Italy; Interdisciplinary Center for Health Sciences, Scuola Superiore Sant’Anna, Piazza Martiri della Libertà, 33, 56127 Pisa, Italy; Department of Cardiology, Aarhus University Hospital, Aarhus, Denmark; Liverpool Centre for Cardiovascular Science, University of Liverpool, Liverpool, United Kingdom; Department of Clinical Medicine, Aalborg University, Aalborg, Denmark; Department of Cardiology, Angiology and Intensive Care Medicine, Deutsches Herzzentrum der Charité, Charité – Universitätsmedizin Berlin, Berlin, Germany; Neurology–Stroke Unit, Saronno Hospital, ASST Valle Olona, Saronno, Italy; Cardiology Department, St. Antonius Ziekenhuis Nieuwegein, Nieuwegein, The Netherlands; Heart Rhythm Management Centre, Universitair Ziekenhuis Brussel, Vrije Universiteit Brussel, Brussels, Belgium; Cardiology Division, Department of Biomedical, Metabolic and Neural Sciences, University of Modena and Reggio Emilia, Policlinico di Modena, Modena, Italy; Molecular and Clinical Sciences Research Institute, City St George’s University of London, London, United Kingdom

**Keywords:** Left atrial appendage closure, Atrial fibrillation, CHAMPION-AF, CLOSURE-AF, Patient selection, Bleeding risk, Competing mortality, Time to benefit

## Abstract

**Aims:**

The near-simultaneous publication of CHAMPION-AF and CLOSURE-AF produced apparently discordant evidence regarding percutaneous left atrial appendage closure (LAAC) for stroke prevention in atrial fibrillation. We aimed to reconcile these findings through a phenotype-based framework and identify the determinants of net clinical benefit.

**Methods and results:**

This narrative review integrates randomized trials, registries, consensus documents, and guidelines, focusing on patient selection, procedural risk, post-implant antithrombotic therapy, endpoint construction, competing mortality, and time to benefit. CHAMPION-AF evaluated LAAC against direct oral anticoagulation in an elective, anticoagulation-eligible population with lower bleeding risk, demonstrating non-inferior thromboembolic protection and less non-procedural bleeding. Conversely, CLOSURE-AF enrolled an older, multimorbid population with substantial renal dysfunction, previous bleeding, and high competing mortality. Although implantation success and stroke rates were comparable between treatment groups, early procedural and antithrombotic-related bleeding, a composite endpoint incorporating procedural events, and limited survival reduced the opportunity for delayed benefit. The highly selective recruitment pathway further limits generalizability. These findings define a continuum from the ‘CHAMPION-like’ patient, with sufficient physiological reserve and life expectancy to realize long-term benefit, to the ‘CLOSURE-like’ patient, in whom frailty and competing risk may outweigh the advantage of appendage exclusion.

**Conclusion:**

Left atrial appendage closure efficacy is not determined by device performance alone but by matching the intervention to patient phenotype and time horizon. Selection should integrate thromboembolic and bleeding risk, frailty, renal function, post-implant treatment tolerance, and expected survival. Future trials require transparent screening data, time-sensitive endpoints, landmark analyses, and competing-risk methods.

## Graphical Abstract

**Graphical Abstract:**
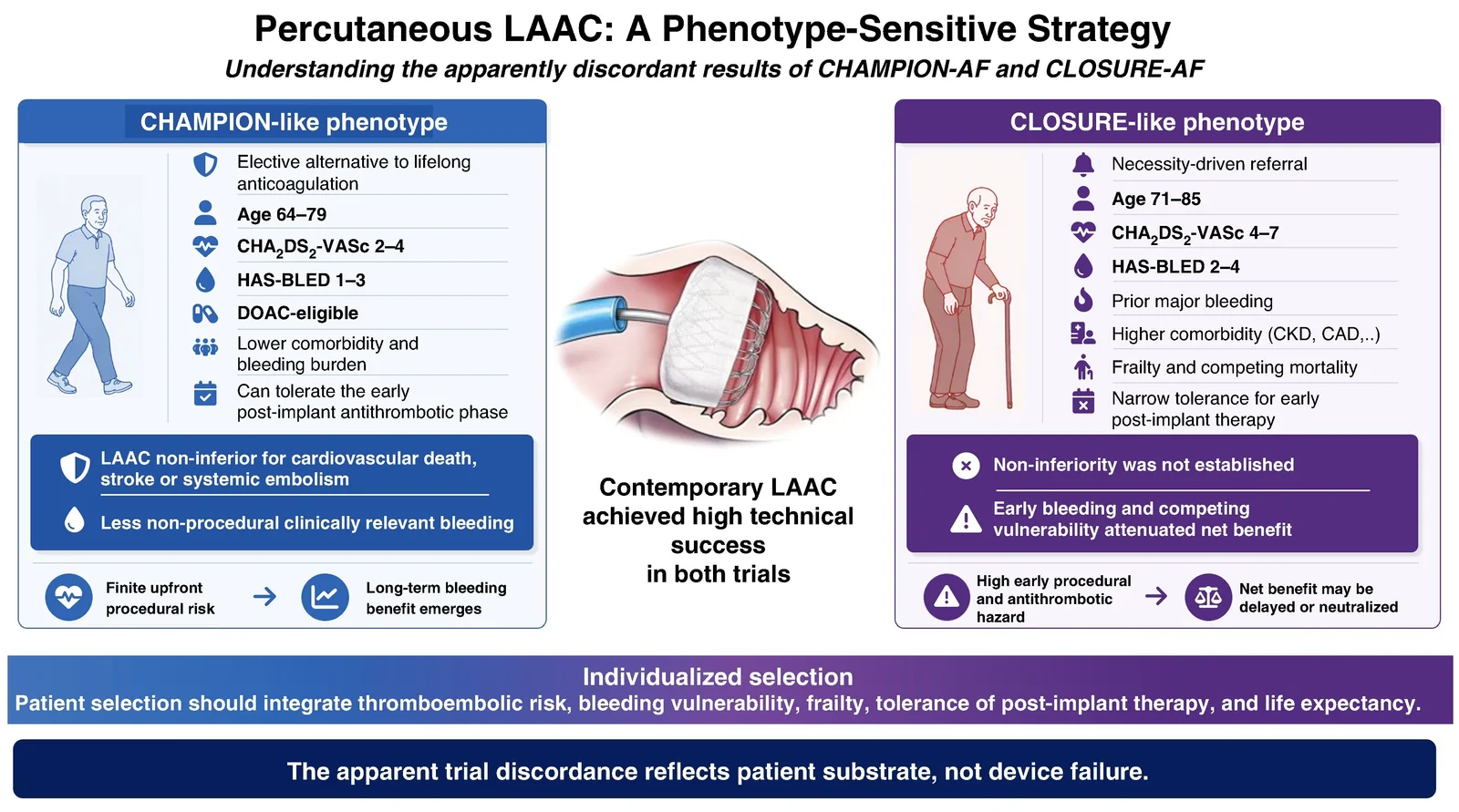
Percutaneous LAAC as a phenotype-sensitive strategy: the CHAMPION-like patient generally has an elective indication, lower bleeding burden, and sufficient tolerance of early post-implant therapy, whereas the CLOSURE-like patient has necessity-driven referral, greater frailty and competing risk, and a narrower margin for early procedural and antithrombotic harm.

## Introduction

Atrial fibrillation (AF) is the most common sustained arrhythmia encountered in clinical practice and represents a major public health challenge because of its close association with cardioembolic stroke, systemic embolus, heart failure, disability, healthcare utilization, and mortality.^1,2^ Contemporary epidemiological data estimate that AF affects approximately 60 million individuals worldwide, with prevalence rising sharply as populations age often surviving with underlying cardiac disease and as detection improves through improved awareness and systematic and digital screening strategies.^3–5^ The clinical relevance of AF derives not only from deleterious haemodynamics but also from the severity of its thromboembolic complications: untreated AF confers an approximately five-fold increase in ischaemic stroke risk, and AF-related strokes are often more disabling and recurrent than strokes of other aetiologies.^6,7^ Oral anticoagulation therefore remains the cornerstone of stroke prevention in AF, with direct oral anticoagulants (DOACs) preferred over vitamin K antagonists in most eligible patients because of their favourable net clinical profile.^4,8^ Yet anticoagulation can be limited by bleeding risk, underuse, discontinuation, perceived frailty, renal dysfunction, previous haemorrhage, and patient preference, leaving a clinical need for non-pharmacological strategies.^8^

Percutaneous left atrial appendage closure (LAAC) emerged from a compelling anatomical and pathophysiological rationale. In nonvalvular AF, thrombus formation tends to be largely localized within the left atrial appendage (LAA), a trabeculated cul-de-sac in which impaired contractility, reduced emptying velocity, endothelial dysfunction, and local hypercoagulability converge to promote thrombogenesis.^9^ Historical series suggested that approximately 90% of atrial thrombi in nonvalvular AF arise in the LAA, while more recent large transoesophageal echocardiography data found extra-appendage left atrial thrombosis to be extremely rare, with virtually all detected thrombi involving the LAA.^10^ This thrombotic substrate provides the mechanistic basis for excluding the LAA from the systemic circulation as a substitute, or complement, to systemic anticoagulation.^11^

Despite a strong biological rationale, the role of LAAC in clinical practice remains incompletely defined and has expanded only gradually.^12^ The 2024 European Society of Cardiology Guidelines confirmed the recommendation for oral anticoagulation as the default strategy for eligible patients at elevated AF-related thromboembolic risk, while percutaneous LAA occlusion remained reserved for selected patients, particularly those with contraindications to long-term anticoagulation, with a Class IIb, level C recommendation reflecting limited randomized evidence and persistent uncertainty regarding patient selection.^6^ Against this background, the near-simultaneous publication of CHAMPION-AF and CLOSURE-AF in the *New England Journal of Medicine* yielded seemingly discordant findings.^13^ In CHAMPION-AF, LAAC was non-inferior to DOAC and was associated with a lower incidence of non-procedural clinically relevant bleeding. By contrast, CLOSURE-AF did not establish non-inferiority relative to physician-directed best medical therapy.^14,15^

This review argues that the paradox is not procedural but phenotypic. CHAMPION-AF and CLOSURE-AF did not test LAAC in the same biological substrate. Rather, they mapped two ends of a clinical continuum: the elective, anticoagulation-eligible, lower-bleeding-risk ‘CHAMPION-like’ patient and the frail, high-ischaemic-risk, high-bleeding-risk, necessity-driven ‘CLOSURE-like’ patient. The central thesis of this narrative review is that the net clinical benefit of LAAC is primarily determined by the interaction between baseline vulnerability, post-implant antithrombotic tolerance, endpoint architecture, and time horizon rather than by implantation success and implanting features. The CHAMPION-like vs. CLOSURE-like framework therefore offers a unifying interpretive key for contemporary LAAC evidence and a practical model for individualized decision-making.

## Trial design and baseline population characteristics

CHAMPION-AF and CLOSURE-AF were both prospective randomized trials evaluating catheter-based LAAC for stroke prevention in AF, yet their hypotheses, comparators, and enrolled populations differed substantially^14,15^ (*Table 1*).

**Table 1:** Comparison of CLOSURE-AF and CHAMPION-AF: study design, baseline population, procedural outcomes, and primary endpoint results

Characteristic	CLOSURE-AF (Landmesser et al., 2026^15^)	CHAMPION-AF (Doshi et al., 2026^14^)
STUDY DESIGN		
Sponsor	German Center for Cardiovascular Research (DZHK)	Boston Scientific
Objective	LAAC vs. physician-directed best medical care in AF patients at high risk of stroke and bleeding	LAAC (Watchman FLX) vs. long-term NOAC in anticoagulation-eligible AF patients
Design	Pragmatic RCT, open-label, blinded outcome assessment	Prospective international RCT, open-label; 3-year analysis (ongoing to 5-year)
Centres/countries	42 centres—Germany	141 centres—16 countries
Enrolment period	March 2018 to May 2024 (62 months)	October 2020 to November 2022 (25 months)
Follow-up analysed	Median 3.0 years (IQR 1.7–4.7)	3 years (Kaplan–Meier)
Comparator	Best medical care: DOAC 85.1%, antiplatelet, or none	Approved NOAC (100%)
Device(s)	Watchman/Watchman FLX; Amplatzer Cardiac Plug/Amulet; LAmbre (bailout)	Watchman FLX exclusively
BASELINE CHARACTERISTICS		
Patients randomized	912 (888 in primary analysis: 446 device/442 medical)	3000 (1499 device/1501 anticoagulation)
Mean age (years)	77.9 ± 7.1	71.7 ± 7.5
Female sex	38.6%	31.9%
CHA_2_DS_2_-VASc score	5.2 ± 1.5	3.5 ± 1.3
HAS-BLED score	3.0 ± 0.9	1.3 ± 0.8
Prior stroke/TIA	33.0%	7.7–7.9% (prior ischaemic stroke without TIA)
Prior major bleeding (BARC ≥3)	> 40%	3.3–3.5%
CKD Stage IV	24.1%	Not specifically reported
Prior AF ablation	Not reported	47.8%
PROCEDURAL OUTCOMES		
Implantation success	98.3%	98.8%
Effective closure (≤3–5 mm leak)	97.6% technical; 96.4% procedural	98.6% at 4-month imaging
Periprocedural complications (≤7 days)	5.7%	1.1%
Post-implant antithrombotic (device group)	DAPT 79.6%; OAC-based 14.8%	NOAC-based 85.0%; DAPT 12.6%
PRIMARY ENDPOINT RESULTS		
Primary endpoint	Composite: stroke + systemic embolism + major bleeding (BARC ≥3) + CV/unexplained death (non-inferiority; margin HR 1.3; RMST analysis)	Efficacy: CV death + stroke + systemic embolism (non-inferiority; margin 4.8 pp) Safety: non-procedure-related bleeding (superiority)
Primary efficacy result	16.8 vs. 13.3/100 pt-years; RMST diff −0.36 yr (95% CI −0.70 to −0.01) *P* = 0.44 → Non-inferiority NOT met	5.7% vs. 4.8% (KM); diff +0.9 pp (95% CI −0.8 to 2.6); HR 1.20 (0.87–1.66) *P* < 0.001 → Non-inferiority MET
Stroke	2.6 vs. 2.7/100 pt-years (virtually identical)	3.6% vs. 2.5% (KM); HR 1.46 (0.94–2.27)
Non-procedure-related bleeding	7.4 vs. 6.2/100 pt-years (no reduction)	10.9% vs. 19.0%; HR 0.55 (0.45–0.67); *P* < 0.001 → LAAC superior
Early vs. late bleeding (device arm)	Early ≤3 months: 9.0% vs. 3.9% (device higher) Late >3 months: 8.1% vs. 11.4% (device lower)	Bleeding benefit accumulates progressively post-endothelialization
Overall verdict	LAAC was NOT non-inferior to best medical care	LAAC was NON-INFERIOR (efficacy) and SUPERIOR (bleeding)

AF, atrial fibrillation; BARC, Bleeding Academic Research Consortium; CKD, chronic kidney disease; CV, cardiovascular; DAPT, dual antiplatelet therapy; DOAC, direct oral anticoagulant; HR, hazard ratio; IQR, interquartile range; ISTH, International Society on Thrombosis and Haemostasis; KM, Kaplan–Meier; LAAC, left atrial appendage closure; NOAC, non-vitamin K antagonist oral anticoagulant; OAC, oral anticoagulation; pt-years, patient-years; RCT, randomized controlled trial; RMST, restricted mean survival time; TIA, transient ischaemic attack.

CHAMPION-AF was an ongoing, international, industry-sponsored randomized trial designed to test whether LAAC with the WATCHMAN FLX device could serve as a reasonable alternative to long-term DOAC therapy in patients with AF who were suitable candidates for anticoagulation. The trial randomized 3000 patients in a 1:1 ratio to LAAC or approved DOAC therapy, with 1499 assigned to the device group and 1501 to anticoagulation. The primary efficacy endpoint was a composite of cardiovascular death, stroke, or systemic embolism at 3 years, while the primary safety endpoint was non-procedure-related bleeding, explicitly separating efficacy from safety.^14^

CLOSURE-AF, by contrast, was a pragmatic, publicly sponsored German multicentre randomized trial testing LAAC against physician-directed best medical care in patients with AF at high risk of both stroke and bleeding, or with contraindications to long-term anticoagulation. A total of 912 patients underwent randomization, with the primary analysis including 446 patients assigned to LAAC and 442 to medical therapy. Unlike CHAMPION-AF, CLOSURE-AF allowed several devices—including Watchman, Watchman FLX, Amplatzer Cardiac Plug, Amulet, and LAmbre—as a bailout option, thereby reflecting real-world practice. The comparator was not uniform DOAC therapy but physician-directed care, although 85.1% of patients in the medical-therapy arm received a DOAC, emphasizing that the control arm represented contemporary optimized medical practice rather than undertreatment.^15^

The most important distinction between the trials lies in patient phenotype. CHAMPION-AF enrolled a relatively elective population: mean age 71.7 years, mean CHA_2_DS_2_-VASc score 3.5, mean HAS-BLED score 1.3, prior ischaemic stroke in approximately 7.6–7.9%, and recent major bleeding as an exclusion criterion. Nearly half had undergone previous AF ablation, reinforcing the impression of a rhythm-management-oriented, procedurally eligible cohort.^14^

CLOSURE-AF enrolled a markedly different population: mean age 77.9 years, mean CHA_2_DS_2_-VASc score 5.2, mean HAS-BLED score 3.0, prior stroke or transient ischaemic attack in 33.0%, history of major BARC 3a–3c bleeding in more than 40%, and Stage IV chronic kidney disease in 24.1%.^15^ These were not simply older versions of the same patients; they represented a different biological and clinical phenotype.

Beyond baseline risk scores, the recruitment process raises important questions regarding external validity. Between March 2018 and May 2024, CLOSURE-AF screened 10 872 patients at 42 German sites and randomized 912 (8.4%); 888 were included in the primary analysis after the exclusion of one site because of failed data-quality control involving 23 patients and one patient who had been erroneously enrolled in another interventional study. Although the original sample-size calculation envisaged 1586 participants, the target was prospectively revised after blinded reassessments of the pooled event rate, initially to 970 patients, and recruitment was subsequently stopped at 912 when updated assumptions suggested adequate—and potentially excessive—statistical power. Thus, the difference from the original target should not be interpreted simply as recruitment failure. Nevertheless, the published materials do not provide a detailed account of why most screened patients were not randomized or whether potentially eligible patients underwent reimbursed LAAC outside the trial. A reimbursement-related negative-selection mechanism is therefore plausible but remains unproven. In the medical-therapy group, 85.1% of patients received a DOAC at treatment initiation, consistent with the protocol-defined use of DOACs as the default therapy when anticoagulation was considered feasible; importantly, patients unsuitable for anticoagulation were not prospectively identified. Renal disease was common: centre-reported renal insufficiency was present in 648 of 881 patients (73.6%), whereas Stage IV chronic kidney disease, defined by an eGFR of 15–29 mL/min/1.73 m^2^, affected 214 of 888 patients (24.1%). Although Stage IV chronic kidney disease was one of the qualifying high-bleeding-risk criteria, the available data do not establish that renal dysfunction was the principal pathway into the trial. More complete reporting of screening exclusions, reasons for non-randomization, routine LAAC outside the trial, and reimbursement status would help clarify the transportability of CLOSURE-AF to contemporary clinical practice.

This distinction recalls the methodological lesson of MITRA-FR and COAPT in secondary mitral regurgitation: apparently discordant procedural trials may be reconciled when the underlying disease substrate, proportionality of risk, and timing of intervention are recognized as central modifiers of treatment effect.^16,17^ Similarly, the opposite outcomes of CHAMPION-AF and CLOSURE-AF should not be interpreted as evidence of different procedural quality and efficacy, but as evidence that LAAC performance depends critically on the substrate to which it is applied.

## Procedural success, post-implant antithrombotic management, and primary endpoints

Both trials confirmed that contemporary LAAC is technically mature. In CHAMPION-AF, device deployment was successful in 1386 patients, corresponding to a 98.8% implantation success rate among attempted procedures, and effective closure with residual leak ≤3 mm was observed in 98.6% of assessed patients at 4 months.^14^ In CLOSURE-AF, implantation was attempted in 421 patients and was successful in 414, yielding an implantation success rate of 98.3%, with technical success in 97.6% and procedural success in 96.4%.^15^ These parallel figures are crucial: the divergence in outcomes cannot be attributed to technical failure of LAAC.

The safety signal, however, differed sharply. CHAMPION-AF reported a low periprocedural complication burden, whereas CLOSURE-AF recorded periprocedural complications in 5.7% of patients within 7 days or before discharge, including major bleeding requiring transfusion, pericardial tamponade, device embolization requiring surgery, procedure-related transient ischaemic attack, peripheral embolism, and two early deaths.^15^ This discrepancy is best understood as an interaction between procedure and frailty rather than between procedure and operator competence. A standardized intervention carries a different biological cost when applied to a 72-year-old DOAC-eligible patient with low haemorrhagic risk or to an octogenarian with prior major bleeding, advanced chronic kidney disease, and competing mortality risks.

Post-implant antithrombotic therapy is the central mechanistic bridge between patient substrate and early harm. In CHAMPION-AF, 85.0% of successfully implanted patients were discharged on DOAC therapy and only 12.6% on dual antiplatelet therapy (DAPT), a strategy tolerated by a population with a mean HAS-BLED score of 1.3.^14^ In CLOSURE-AF, 79.6% of device-treated patients received DAPT as initial therapy, reflecting the concern that many were poor anticoagulation candidates; yet this compromise exposed a frail, bleeding-prone population to an antithrombotic regimen during the thrombogenic endothelialization window.^15^ The inversion of haemorrhagic risk in CLOSURE-AF was strikingly evident: early major bleeding was higher in the LAAC arm (9.0% vs. 3.9% in the first months), whereas the later trajectory shifted towards 8.1% vs. 11.4% in favour of LAAC after the endothelialization phase.^15^

Endpoint design amplified these differences. CHAMPION-AF separated efficacy and safety: LAAC met non-inferiority for cardiovascular death, stroke, or systemic embolism at 3 years, with event rates of 5.7% vs. 4.8%, and was superior for non-procedure-related bleeding (10.9% vs. 19.0%; hazard ratio 0.55; p_superiority_<0.001).^14^ CLOSURE-AF used a single time-to-event composite combining stroke, systemic embolism, major bleeding, and cardiovascular or unexplained death, and LAAC failed non-inferiority, with primary endpoint incidence of 16.8 vs. 13.3 events per 100 patient-years.^15^ Yet stroke rates were virtually identical—2.6 vs. 2.7 per 100 patient-years—underscoring that CLOSURE-AF did not demonstrate failure of stroke prevention, but rather temporal penalization of LAAC by early bleeding events and competing vulnerability embedded in a composite endpoint that weighted an early major bleed similarly to later thromboembolic protection.^15^

The endpoint architecture deserves equally cautious interpretation. By incorporating procedure-related bleeding into the primary composite, CLOSURE-AF counted the one-time, front-loaded hazard of implantation together with recurrent late events while applying a non-inferiority margin corresponding to a hazard ratio of 1.30. The 18 procedure-related major bleeds in the LAAC group materially influenced the comparison; in a post-hoc scenario excluding these events, LAAC would have satisfied non-inferiority vs. physician-directed therapy. This does not invalidate the prespecified primary result, but it shows that the negative conclusion is sensitive to how early procedural harm is weighted relative to delayed stroke and bleeding prevention.^15^

## Defining the archetypes: the CHAMPION-like and the CLOSURE-like patient

The CHAMPION-like and CLOSURE-like phenotypes should be understood as archetypes rather than rigid categories. The CHAMPION-like patient is typically 70–75 years old; has nonvalvular AF, a CHA_2_DS_2_-VASc score of 4 or less, a HAS-BLED score below 3, and preserved eligibility for DOAC therapy; and seeks LAAC as an elective alternative to lifelong anticoagulation because of preference, adherence concerns, lifestyle considerations, or the desire to reduce cumulative bleeding exposure. In this phenotype, the patient can tolerate the short-term antithrombotic ‘entry cost’ required after implantation, allowing the long-term mechanical advantage of LAA exclusion to emerge. CHAMPION-AF therefore supports LAAC as a plausible elective strategy in selected anticoagulation-eligible patients.^14,18^

The CLOSURE-like patient occupies the opposite pole: age around 77–80 years, CHA_2_DS_2_-VASc score ≥5, HAS-BLED score ≥3, prior major bleeding, chronic kidney disease, frailty, competing mortality risks, and a necessity-driven referral because standard anticoagulation appears problematic. In this phenotype, LAAC may remain biologically attractive because thrombi still arise predominantly from the LAA. However, the relative contribution of the LAA to cardioembolic stroke may be reduced in these patients, as multiple cardiovascular comorbidities can give rise to alternative sources of thromboembolism. Moreover, the early hazards of access, anaesthesia, pericardial complications, DAPT, bleeding recurrence, and limited physiological reserve may reduce or delay net clinical benefit.^10,15,19^

Between these poles lies a clinical continuum rather than a binary decision rule, in which the expected net clinical benefit of LAAC widens or narrows according to age, thromboembolic risk, bleeding risk, renal function, frailty, post-implant therapy tolerance, and life expectancy. In this framework, LAAC, similarly to other interventional approaches, cannot be simply labelled as effective or ineffective; it is phenotype-sensitive. Its optimal use requires matching the procedure to the patient’s biological reserve and to the antithrombotic strategy that the patient can safely sustain during the endothelialization phase.

## Evidence from trials enrolling comparable CHAMPION-like populations

The CHAMPION-like phenotype identifies patients in whom percutaneous LAAC is not primarily a rescue therapy for failed anticoagulation, but an elective alternative to long-term oral anticoagulant exposure. Its defining features are comparatively modest bleeding risk, preserved eligibility for DOACs, younger age, and an intermediate thromboembolic profile, typically reflected by HAS-BLED scores below 3, CHA_2_DS_2_-VASc scores around or below 4, fewer comorbidities, and mean age below 75 years.

In this phenotype, the central clinical question is not whether LAAC can be performed when anticoagulation is impossible, but whether a device-based strategy can reproduce the anti-thromboembolic protection of anticoagulation while progressively reducing the cumulative burden of bleeding.

The foundational evidence for this concept began with PROTECT AF, the first randomized trial to compare LAAC using the first-generation WATCHMAN device with long-term anticoagulation with warfarin in patients with nonvalvular AF. The trial enrolled 707 patients, with a mean CHADS_2_ score of approximately 2.2, and demonstrated non-inferiority of LAAC for the composite of stroke, systemic embolism, and cardiovascular or unexplained death.^20^ PROTECT AF was not a contemporary DOAC-era trial, and its procedural safety profile reflected early-generation technology and the beginning of the operator learning curve; primary safety events were more frequent in the device arm, largely because of pericardial effusion and procedure-related bleeding. Nevertheless, the trial established the central biological premise of LAAC: after successful endothelialization and discontinuation of warfarin, protection from LAA thromboembolism could be maintained without indefinite systemic anticoagulation.^20^

PREVAIL was designed to provide additional evidence on LAAC safety profile, with more rigorous procedural standards and broader operator experience. The trial randomized 407 patients, with a mean CHADS_2_ score of 2.6 and CHA_2_DS_2_-VASc score close to 3.8–3.9, to Watchman implantation or warfarin therapy.^16^ PREVAIL did not meet all prespecified non-inferiority criteria for the first co-primary efficacy endpoint, largely because of unexpectedly low event rates and statistical fragility rather than a clear signal of clinical inferiority. However, it did meet non-inferiority for late ischaemic stroke or systemic embolism beyond the immediate periprocedural period, thereby reinforcing the mechanistic hypothesis that, once procedural risk is traversed, appendage exclusion provides durable thromboembolic protection. Long-term pooled analyses of PROTECT AF and PREVAIL further demonstrated progressively increasing reductions in haemorrhagic stroke, disabling or fatal stroke, and mortality with extended follow-up.^21^

Amulet IDE extended this maturation phase by providing device-vs.-device randomized evidence. In this trial of 1878 patients, the Amulet occluder was compared with the first-generation Watchman device in patients at increased stroke risk, with mean CHA_2_DS_2_-VASc scores of 4.5–4.7 and HAS-BLED scores of 3.2–3.3.^22^ Amulet was non-inferior to Watchman for the composite safety endpoint and for ischaemic stroke or systemic embolism and provided superior anatomical closure at 45 days. However, procedure-related complications were more frequent with Amulet, particularly pericardial effusion and device embolization, underscoring that device selection and operator experience remain determinants of net benefit even in lower-risk or elective candidates.

The next stage in the CHAMPION-like evidence pathway was device refinement. PINNACLE FLX was a prospective, multicentre, non-randomized FDA study evaluating the next-generation Watchman FLX device in 400 patients with nonvalvular AF, mean age 73.8 years, mean CHA_2_DS_2_-VASc score 4.2, and mean HAS-BLED score 2.0.^23^ The primary safety endpoint occurred in only 0.5% of patients, and effective appendage closure at 12 months was achieved in essentially all evaluable patients, suggesting that next-generation conformability, recapturability, and sealing performance had shifted LAAC from an experimental alternative towards a reproducible elective procedure.

The DOAC-era elective strategy was directly tested in OPTION, which randomized 1600 patients after AF ablation to LAAC or continued oral anticoagulation.^24^ This trial enrolled a distinctly CHAMPION-like population: mean age 69.6 years, mean CHA_2_DS_2_-VASc score 3.5, and mean HAS-BLED score 1.2. At 36 months. LAAC was superior for non-procedure-related major or clinically relevant nonmajor bleeding (8.5% vs. 18.1%) while remaining non-inferior for the composite of death from any cause, stroke, or systemic embolism. OPTION is particularly relevant because it addressed a common contemporary dilemma: patients who undergo successful ablation often remain guideline-eligible for anticoagulation since stroke risk is determined by clinical risk factors rather than apparent rhythm control alone.^25^

Across CHAMPION-like trials, the consistent message is that LAAC should be interpreted as an elective, time-dependent strategy. The initial procedural and post-implant phase carries a finite hazard, whereas the bleeding advantage becomes progressively more apparent once the device is endothelialized and systemic anticoagulation is discontinued. In the CHAMPION-like phenotype, LAAC is best framed as a planned risk-reallocation intervention: it exchanges a limited upfront procedural and short-term antithrombotic risk for a progressive reduction in lifelong anticoagulant-related bleeding burden. The characteristics of CHAMPION-like LAAC studies are listed in *Table 2*.

**Table 2:** Characteristics of CHAMPION-like LAAC studies

Study (first author, year)	Design/comparator	*n*	Population risk profile	Device(s)	Post-implant antithrombotic regimen	Main outcome	Interpretation within framework
PROTECT AF (Holmes et al., 2009)	Randomized non-inferiority trial; LAAC vs. warfarin	707	CHADS_2_ 2.2; moderate stroke risk; warfarin-eligible	Watchman first generation	Warfarin + aspirin after implantation, then antiplatelet strategy after closure confirmation	Non-inferior to warfarin for stroke/systemic embolism/cardiovascular or unexplained death; higher early procedural complications reflecting first-generation technology and learning curve	CHAMPION-like prototype: elective anticoagulation alternative in moderate-risk, anticoagulation-eligible patients
PREVAIL (Holmes et al., 2014)	Randomized trial; LAAC vs. warfarin	407	CHADS_2_ 2.6; CHA_2_DS_2_-VASc ∼3.8–3.9; anticoagulation-eligible	Watchman first generation	Warfarin-based post-implant regimen followed by antiplatelet therapy	Did not meet all first co-primary efficacy non-inferiority criteria because of low event rates, but met late ischaemic stroke/systemic embolism criterion; improved procedural safety vs. PROTECT AF	Supports time-dependent benefit after early procedural risk has been traversed
PINNACLE FLX (Kar et al., 2021^23^)	Prospective multicentre single-arm FDA study	400	Mean age 73.8 years; CHA_2_DS_2_-VASc 4.2; HAS-BLED 2.0	Watchman FLX	Short-term DOAC + aspirin, then antiplatelet-based strategy according to protocol	Primary safety endpoint 0.5%; effective closure at 12 months in essentially all evaluable patients	Device-maturation study showing reproducible safety in an elective, lower-bleeding-risk phenotype
Amulet IDE (Lakkireddy et al., 2021)	Randomized device-vs.-device trial; Amulet vs. Watchman	1878	CHA_2_DS_2_-VASc ∼4.5–4.7; HAS-BLED ∼3.2–3.3; borderline CHAMPION-like/closure continuum	Amulet vs. Watchman first generation	Amulet: mainly DAPT-based regimen; Watchman: anticoagulation-based regimen according to device protocol	Amulet non-inferior for safety/effectiveness and superior for anatomical closure; more procedure-related complications with Amulet	Relevant to CHAMPION-like evidence as device–platform validation rather than anticoagulation–substitution trial
OPTION (Wazni et al., 2024)	Randomized trial after AF ablation; LAAC vs. oral anticoagulation	1600	Mean age 69.6 years; CHA_2_DS_2_-VASc 3.5; HAS-BLED 1.2	Watchman FLX	Post-implant antithrombotic therapy according to device protocol, then discontinuation of long-term OAC when appropriate	LAAC superior for non-procedure-related major or clinically relevant nonmajor bleeding and non-inferior for death/stroke/systemic embolism at 36 months	Strong CHAMPION-like model: elective LAAC in low-bleeding-risk patients otherwise remaining eligible for OAC after ablation
CHAMPION-AF (Doshi et al., 2026^14^)	International randomized trial; LAAC vs. NOAC therapy	3000	Mean age 71.7 years; CHA_2_DS_2_-VASc 3.5; HAS-BLED 1.3; recent major bleeding excluded	Watchman FLX	Most patients discharged on NOAC-based therapy (85.0%); small proportion on DAPT (12.6%); then single antiplatelet strategy	Non-inferior for cardiovascular death/stroke/systemic embolism at 3 years; superior for non-procedure-related bleeding (10.9% vs. 19.0%)	Archetypal CHAMPION-like trial: LAAC as elective, time-dependent bleeding–reduction strategy

CHAMPION-like studies are characterized by comparatively lower bleeding risk, preserved eligibility for oral anticoagulation, generally younger age than CLOSURE-like cohorts, and an elective strategy in which LAAC is evaluated as an alternative to long-term anticoagulant exposure rather than as rescue therapy. AF, atrial fibrillation; DAPT, dual antiplatelet therapy; DOAC, direct oral anticoagulant; LAAC, left atrial appendage closure; NOAC, non-vitamin K antagonist oral anticoagulant; OAC, oral anticoagulation.

## Evidence from trials enrolling comparable CLOSURE-like populations

The CLOSURE-like phenotype occupies a fundamentally different clinical space. These patients are older, frailer, and more comorbid, with CHA_2_DS_2_-VASc scores typically at or above 5, HAS-BLED scores at or above 3, frequent prior bleeding, renal dysfunction, contraindications to oral anticoagulation, and limited physiological reserve. In this setting, LAAC is rarely an elective strategy; it is considered because both available options are unsatisfactory: anticoagulation may be unsafe or impossible, whereas withholding anticoagulation leaves the patient exposed to a high risk of cardioembolic stroke.^15^ The evidence base in this phenotype is more heterogeneous, more registry-driven, and more sensitive to the antithrombotic regimen used after implantation.

The ASAP study was the first systematic prospective experience specifically designed for patients considered ineligible for warfarin. It enrolled 150 patients with nonvalvular AF, a mean CHADS_2_ score of 2.8, and a bleeding-risk profile approximating HAS-BLED 3.0, using DAPT rather than warfarin after first-generation Watchman implantation.^26^ The observed ischaemic stroke rate was 1.7% per year, substantially lower than the expected rate of approximately 7.3% per year based on baseline thromboembolic risk, corresponding to an estimated 77% reduction.^26^ ASAP was not randomized and therefore could not exclude selection bias, but it established a crucial principle for the CLOSURE-like population: appendage closure could be technically feasible and potentially protective even when conventional oral anticoagulation was not an option.

EWOLUTION expanded this experience into a large real-world European registry enrolling 1025 patients, with a mean CHA_2_DS_2_-VASc score 4.5 and a mean HAS-BLED score 2.3, and 62% deemed unsuitable for oral anticoagulant therapy.^27^ Device deployment was successful in 98.5% of patients, with no or minimal residual flow in nearly all successfully implanted cases. Subsequent EWOLUTION analyses confirmed that competing mortality risk was substantial; 2-year mortality was driven less by the device itself than by age, comorbidity, renal impairment, heart failure, and bleeding history.^28^ This observation is central to the CLOSURE-like framework: procedural success does not automatically translate into large absolute net benefit when non-cardiovascular mortality and frailty dominate the prognosis.

PRAGUE-17 provided the first randomized comparison of LAAC against DOACs in a bilaterally high-risk population. The trial enrolled 402 patients with a mean CHA_2_DS_2_-VASc score 4.7 and a mean HAS-BLED score of 3.0–3.1, including patients with prior bleeding requiring intervention or hospitalization, cardioembolic events despite anticoagulation, or combined high thrombotic and bleeding risk.^29^ Left atrial appendage closure was non-inferior to DOAC therapy for a broad composite endpoint including stroke, transient ischaemic attack, systemic embolism, cardiovascular death, clinically significant bleeding, and procedure- or device-related complications. PRAGUE-17 is therefore best viewed as proof-of-concept that LAAC can be an alternative to DOACs in selected high-risk patients, rather than definitive evidence that it should replace optimized medical therapy across the entire CLOSURE-like spectrum.

ASAP-TOO attempted to address the most difficult subgroup: patients with AF considered unsuitable for oral anticoagulation, randomized to LAAC or non-anticoagulant medical therapy (antiplatelet or nothing) using Watchman FLX technology in a population with a mean CHA_2_DS_2_-VASc score of 4.7 and a HAS-BLED score of 3.2.^30^ However, enrolment and randomization rate decreased through 2020, and with the added challenge of COVID-19, enrolment was stopped with 484 patients enrolled, out of the planned 888, leaving the trial underpowered for definitive clinical conclusions. ASAP-TOO reinforces the methodological difficulty of studying the CLOSURE-like phenotype: the very complex patients who most need evidence are often the hardest to randomize, sustain in follow-up, and treat with standardized post-implant antithrombotic therapy. Although underpowered, the ASAP-TOO exploratory data are consistent with an ability of LAAC to reduce the risk of stroke or cardiovascular death, but at the cost of an increased risk of bleeding, particularly during the early post-procedural period.

Taken together, CLOSURE-like studies demonstrate that LAAC can reduce stroke risk in patients who cannot tolerate conventional anticoagulation, but they also reveal that post-implant therapy is the critical bottleneck. Consensus documents emphasize that antithrombotic therapy after LAAC must balance device-related thrombosis against bleeding vulnerability, yet this balance is most precarious particularly in frail, elderly, renally impaired, or previously bleeding patients.^31,32^ Evidence in end-stage renal disease and very elderly populations confirms that implantation can be technically feasible, but clinical outcomes remain strongly conditioned by comorbidity, competing mortality, and periprocedural vulnerability.^33,34^ Therefore, in the CLOSURE-like phenotype, LAAC should not be framed simply as ‘less anticoagulation’. It is a strategy whose success depends on whether the patient can safely traverse the procedural and early antithrombotic window—and when that window is narrow, the early hazard may or outweigh the delayed benefit of reduced bleeding. The characterization of thromboembolic and bleeding risk in all the trials that evaluated an antithrombotic strategy based on LAAC implant is shown in *Figure 1*, and all related details are reported in *Table 2* and *Table 3*.

**Figure 1:**
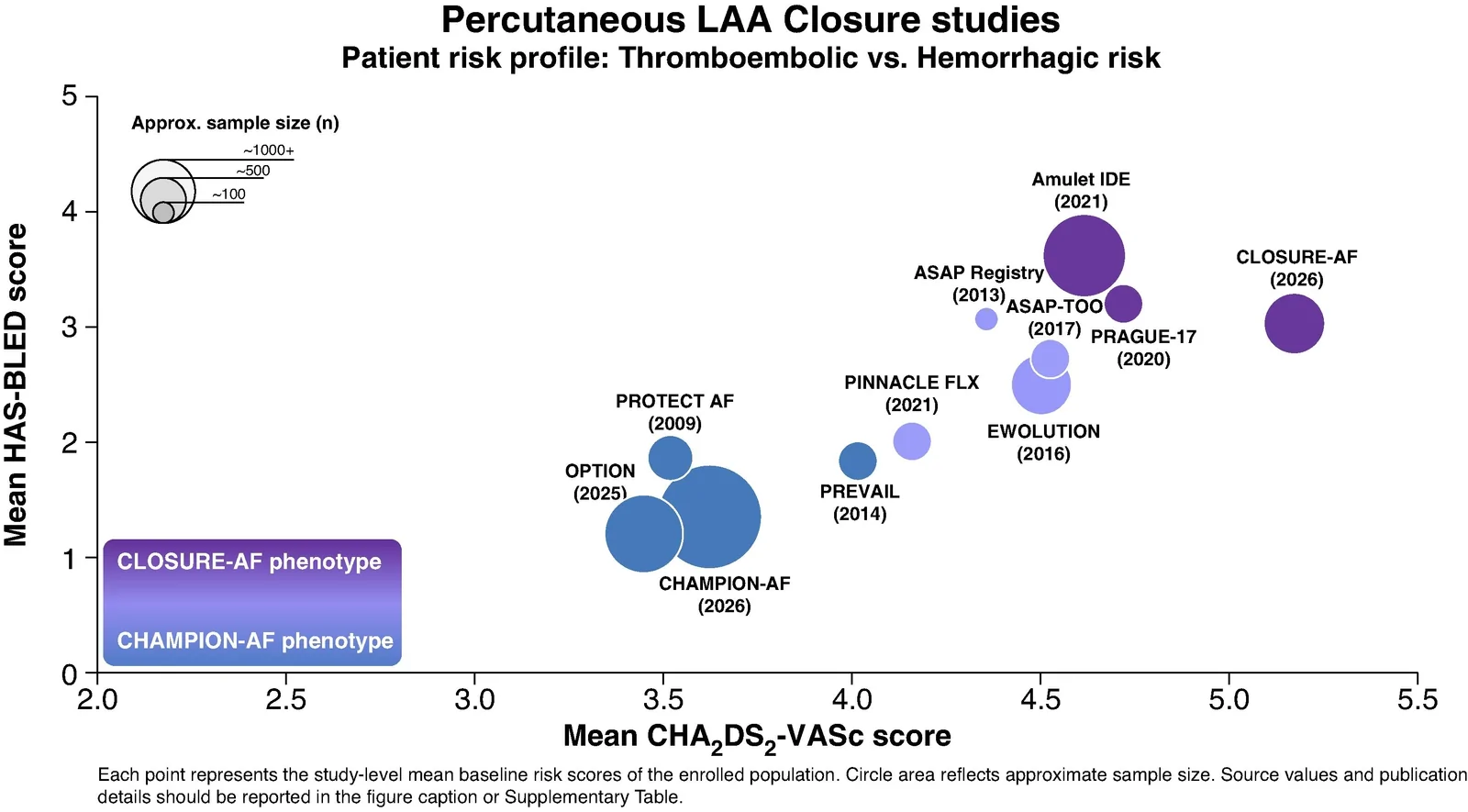
Risk-profile continuum across percutaneous left atrial appendage closure studies. Each point represents the study-level mean baseline CHA_2_DS_2_-VASc and HAS-BLED scores of the enrolled population; circle area approximates sample size. Blue shading denotes the CHAMPION-AF-like phenotype and purple shading the CLOSURE-AF-like phenotype. Source values and publication details are reported in *Tables 2* and *3*.

**Table 3:** Characteristics of CLOSURE-like LAAC studies

Study (first author, year)	Design/comparator	*n*	Population risk profile	Device(s)	Post-implant antithrombotic regimen	Main outcome	Interpretation within framework
ASAP (Reddy et al., 2013^26^)	Prospective non-randomized registry in warfarin-ineligible patients	150	CHADS_2_ 2.8; HAS-BLED ∼3.0; mean CHA_2_DS_2_-VASc score: 4.4 contraindication to warfarin	Watchman first generation	DAPT without warfarin after implantation	Observed ischaemic stroke rate 1.7%/year vs. expected ∼7.3%/year; estimated 77% reduction	Early proof that LAAC may be feasible when conventional anticoagulation is not possible, but non-randomized
EWOLUTION (Boersma et al., 2016^27^)	Large prospective real-world European registry	1025	CHA_2_DS_2_-VASc 4.5; HAS-BLED reported around 2.3–3.3 across analyses; 62% unsuitable for OAC	Watchman first generation	Highly variable post-implant regimens, including DAPT, OAC, single antiplatelet therapy, or no therapy	Implant success 98.5%; low residual flow; subsequent analyses showed outcomes strongly conditioned by age, renal dysfunction, HF, bleeding history, and competing mortality	Real-world CLOSURE-like evidence: procedural feasibility does not eliminate comorbidity-driven prognosis
PRAGUE-17 (Osmancik et al., 2020^29^)	Randomized trial; LAAC vs. DOACs in high-risk AF	402	CHA_2_DS_2_-VASc 4.7; HAS-BLED ∼3.0–3.1; prior bleeding or high thromboembolic/bleeding risk	Amulet or Watchman platforms	DAPT after LAAC followed by aspirin; DOAC in control arm, mostly apixaban	LAAC non-inferior to DOACs for a broad composite including stroke/TIA/systemic embolism, cardiovascular death, clinically significant bleeding, and device/procedure complications	Randomized CLOSURE-like proof-of-concept, but small sample and broad endpoint limit precision
ASAP-TOO (Holmes et al., 2024; Reddy et al., 2026)	Randomized trial 2:1 in patients unsuitable for OAC; prematurely terminated/under-enrolled; LAAC vs. non-anticoagulant therapy	484 enrolled	CHA_2_DS_2_-VASc ∼4.7; HAS-BLED ∼3.2; non-anticoagulable population	Watchman FLX	Non-OAC post-implant regimen, generally antiplatelet-based according to protocol/bleeding risk or no therapy	Underpowered because of limited enrolment; no definitive clinical efficacy conclusion	Illustrates the difficulty of generating randomized evidence in the highest-need CLOSURE-like subgroup
CLOSURE-AF (Landmesser et al., 2026^15^)	Pragmatic multicentre randomized trial; LAAC vs. physician-directed best medical care	912 randomized; 888 in primary analysis	Mean age 77.9 years; CHA_2_DS_2_-VASc 5.2; HAS-BLED 3.0; prior stroke/TIA 33%; major bleeding >40%; CKD Stage IV 24.1%	Watchman, Watchman FLX, Amplatzer Cardiac Plug, Amulet; LAmbre bailout	DAPT recommended for at least 3 months; 79.6% initially received DAPT; medical arm mostly DOAC (85.1%)	Did not meet non-inferiority for stroke/systemic embolism/major bleeding/cardiovascular or unexplained death; stroke similar, but early bleeding and competing vulnerability penalized LAAC	Archetypal CLOSURE-like trial: high-risk substrate and early post-implant hazard can neutralize delayed benefit

CLOSURE-like studies are characterized by older age, higher thromboembolic and bleeding risk, frailty/comorbidity, contraindication or unsuitability for oral anticoagulation, and greater vulnerability to procedural and early post-implant antithrombotic hazards. CKD, chronic kidney disease; DAPT, dual antiplatelet therapy; DOAC, direct oral anticoagulant; HF, heart failure; LAAC, left atrial appendage closure; OAC, oral anticoagulation; TIA, transient ischaemic attack.

## Clinical implications: patient selection and the CHAMPION/CLOSURE continuum

The CHAMPION-like vs. CLOSURE-like framework should be understood as a pragmatic clinical continuum for patient selection. At one end are patients resembling the CHAMPION-AF population: relatively younger, anticoagulation-eligible individuals with moderate thromboembolic risk, low bleeding burden, and sufficient life expectancy to benefit from the delayed reduction in non-procedure-related bleeding after LAAC.^14^ At the opposite end are CLOSURE-like patients: older, multimorbid, high-risk individuals with substantially higher CHA_2_DS_2_-VASc and HAS-BLED scores, in whom procedural hazards, competing mortality, and limited time to benefit may attenuate or abolish the theoretical advantage of appendage exclusion.^15^

This distinction has immediate practical consequences. Left atrial appendage closure appears most appropriate when the expected cumulative burden of anticoagulation-related bleeding exceeds the upfront procedural risk and when the patient has a life expectancy long enough for this trade-off to mature. Conversely, LAAC should be approached with caution when frailty, renal dysfunction, recurrent bleeding, advanced age, or limited functional reserve increase the likelihood that early complications will dominate the clinical trajectory. This interpretation is consistent with the 2024 ESC Guidelines, which maintain percutaneous LAA occlusion as a Class IIb option in patients with AF and contraindications to long-term anticoagulant treatment.^35^

This consideration can be formalized as time to benefit. Left atrial appendage closure imposes an immediate procedural and post-implant antithrombotic risk, whereas its potential advantages accrue only after the early hazard has been traversed and anticoagulant exposure has been reduced over time. In CLOSURE-AF, all-cause mortality was 13.5 and 14.8 per 100 patient-years in the medical-therapy and LAAC groups, respectively—rates unlikely to be explained by age, CHA_2_DS_2_-VASc, or HAS-BLED alone and suggestive of severe cardiovascular and non-cardiovascular comorbidity not fully captured by conventional scores. The published dataset does not permit attribution to specific conditions such as advanced cancer; nevertheless, the magnitude of competing mortality makes projected survival and frailty more relevant than chronological age alone. A patient should therefore be considered for LAAC only when expected survival is sufficiently long for delayed benefit to outweigh the upfront procedural and antithrombotic cost.^13^

Shared decision-making is therefore not a rhetorical addition but the central clinical act. The decision should integrate age, comorbidity burden, CHA_2_DS_2_-VA/CHA_2_DS_2_-VASc score, structured bleeding-risk assessment and correction of modifiable bleeding risk factors, life expectancy, anatomical suitability, post-implant antithrombotic tolerance, and patient preferences. The debate on expanding indications after CHAMPION-AF appropriately emphasizes case-by-case selection rather than universal substitution of DOACs by LAAC.^18^ In this setting, a graphic visualization of the CHAMPION/CLOSURE continuum may help clinicians communicate uncertainty: patients can be shown how the balance between procedural risk, anticoagulant exposure, bleeding susceptibility, and time horizon shifts across phenotypes. Left atrial appendage closure is elective in the CHAMPION-like patient, potentially reasonable after transparent discussion; it is cautionary in the CLOSURE-like patient, where device availability must not replace individualized risk–benefit judgement.

## Methodological implications: endpoint design and future trial requirements

The divergent results of contemporary LAAC trials highlight a central methodological lesson: patient phenotype is not a secondary covariate but the dominant confounding variable across the entire evidence base. CHAMPION-AF and CLOSURE-AF did not test the same intervention in biologically equivalent populations; they tested the same procedural concept across markedly different substrates, with differences in age, bleeding risk, comorbidity, post-implant regimens, device strategy, and competing mortality.^14,15^ Consequently, future trials should not merely report baseline CHA_2_DS_2_-VA/CHA_2_DS_2_-VASc and HAS-BLED scores; they should prospectively stratify by these scores, prespecify interaction testing, and define clinically meaningful phenotype strata before randomization.

Accordingly, future LAAC trials should report complete screening logs, reasons for non-randomization, treatment received outside the trial, reimbursement status, and detailed competing comorbidities, including active malignancy and advanced organ failure. Prespecified landmark analyses separating procedural from non-procedural events, together with restricted mean event-free survival and competing-risk approaches, would better quantify when net benefit begins to emerge.

Endpoint construction is equally critical. Left atrial appendage closure is an intervention with an asymmetric risk profile: it concentrates hazard early—through access-site complications, pericardial effusion, device embolization, device-related thrombosis, and periprocedural bleeding—while its potential benefit emerges later through reduced exposure to long-term anticoagulation. Conventional time-to-first-event composite endpoints may therefore penalize LAAC for early events without adequately weighting the cumulative long-term reduction in spontaneous bleeding. Future LAAC studies should go further by incorporating time-weighted composite endpoints, recurrent-event analyses, restricted mean survival time, bleeding burden rather than time to first bleeding event, and competing-risk methods, particularly in elderly or frail populations where non-cardiovascular death may obscure treatment effects.

The analogy with transcatheter mitral repair trials is instructive. The apparent discordance between MITRA-FR and COAPT was ultimately understood through differences in patient selection, proportionality of mitral regurgitation, ventricular substrate, and endpoint design.^16,17^

A standardized phenotyping protocol should include thromboembolic risk, bleeding history, AF recurrence, atrial fibrosis, atrial substrate in terms of ‘atrial cardiomyopathy’^36^ AF burden, frailty, renal function, prior stroke, LAA anatomy, antithrombotic feasibility, and projected life expectancy taking into account the entire spectrum of comorbidities and disabilities. Without such harmonization, future studies risk repeating the same interpretive ambiguity: a technically successful procedure may appear beneficial, neutral, or harmful depending less on device performance than on the patient phenotype into which it is implanted.

## Future directions: devices, post-implant regimens, and personalized selection

The future of LAAC will depend on whether technological refinement can reduce the early procedural and post-implant burden sufficiently to expand net clinical benefit without encouraging indiscriminate use. Next-generation devices are moving in this direction. Watchman FLX Pro incorporates a fluoropolymer-coated fabric surface, additional radiopaque markers, and an expanded sizing range, with preclinical evidence suggesting reduced thrombogenicity, inflammation, platelet binding, and faster tissue coverage compared with earlier Watchman platforms.^9^ Similarly, dual-seal designs such as Amulet aim to improve immediate closure and reduce residual leaks, although device-specific trade-offs regarding pericardial effusion, device-related thrombus, and post-implant antithrombotic needs remain clinically relevant.

Equally important is the evolution of post-implant pharmacological treatment. The traditional sequence of short-term anticoagulation or DAPT followed by aspirin was inherited from pivotal trial protocols rather than optimized for contemporary high-bleeding-risk populations. Consensus documents emphasize that post-implant treatment should be individualized, especially because some patients referred for LAAC may bleed even with single antiplatelet therapy.^31,32,37^ Emerging randomized data, including the ANDES, ASPIRIN-LAAO, SAFE-LAAC, RECORD-III, ARMYDA-AMULET, and SIMPLAAFY trials, are central to defining whether device endothelialization can be achieved with less pharmacological exposure. Evidence on single antiplatelet therapy after Amulet implantation further supports testing simplified regimens in carefully phenotyped patients.^38^

Personalized selection will also be transformed by imaging, artificial intelligence, and machine learning. Pre-procedural computed tomography and *trans*-oesophageal echocardiography already support anatomical sizing, leak prediction, and exclusion of thrombus^39–41^; future algorithms may integrate LAA morphology, atrial cardiomyopathy markers, bleeding history, frailty scores, renal function, medication adherence, and competing risk of mortality into a CHAMPION-like vs. CLOSURE-like probability score. Prospective registries should therefore collect rigorous baseline phenotyping rather than procedural success alone.

The OPTION trial provides a model for the CHAMPION-like strategy, showing that LAAC after AF ablation reduced non-procedure-related bleeding while remaining non-inferior to anticoagulation for death, stroke, or systemic embolism.^24^ Early feasibility experience with simultaneous pulsed-field ablation and LAAC suggests that a combined approach is also feasible in selected patients with suitable anatomy.^42,43^ Yet the cautionary perspective remains essential: as Widimský argued after CLOSURE-AF, structural cardiology must avoid transforming a promising structural intervention into an overused procedure unsupported by patient-specific benefit.^19^

## Conclusions

Percutaneous LAAC is an effective strategy for stroke prevention in selected patients with nonvalvular AF, but its net clinical success depends on the biological and clinical substrate in which the device is implanted. The central question is no longer whether the appendage can be closed safely and effectively in anatomical terms, since contemporary devices achieve high procedural success, and randomized trials have shown that appendage exclusion can approximate the protection offered by anticoagulation in selected populations. The key clinical question is not whether LAAC is intrinsically beneficial, but how to individualize treatment according to each patient’s thromboembolic risk, bleeding risk, procedural risk, and life expectancy to maximize net clinical benefit.

CHAMPION-AF and CLOSURE-AF should not be read as contradictory trials, but rather as complementary studies positioned at different points along the same clinical continuum.

The CHAMPION-like/CLOSURE-like framework offers a unifying interpretive lens for the LAAC literature, reconciling earlier warfarin-era trials, PRAGUE-17, OPTION, registry data, and guideline caution within a single patient-centred model. In practical terms, LAAC works best when procedural risk is relatively low, life expectancy is meaningful, anticoagulation-related harm is plausible, and post-implant therapy is tolerable. It becomes uncertain when frailty, extreme bleeding vulnerability, renal disease, recurrent hospitalization, or limited survival shorten the time available for benefit. Future trials must stratify by phenotype before testing devices, incorporate time-weighted endpoints, and collect rigorous baseline phenotyping. The expansion of LAAC indications should proceed through accuracy rather than volume.

Moreover, trials like ELAPSE are evaluating LAAC in a different clinical setting—not as an alternative to anticoagulation, but as an adjunct to continued oral anticoagulant therapy in patients with AF who remain at high risk of recurrent thromboembolism, including those with a previous ischaemic stroke despite adequate DOAC treatment, while LAAOS-4 explores LAAC in high-risk post-stroke populations.^44^

The future challenge is therefore not simply to prove that LAAC works, but to determine how best to individualize its use according to each patient’s thromboembolic risk, bleeding vulnerability, and life expectancy. In this sense, LAAC may become a paradigm of tailored medicine in structural cardiology, where patient risk profile—not device availability—drives therapeutic choice.

## Data Availability

No new data were generated or analysed in support of this review. All data discussed, including the study-level values presented in Tables 1–3 and Figure 1, are derived from previously published studies cited in the reference list.
